# Chinese junior high school students’ family background and learning engagement: the parallel mediations of self-educational expectations and parental educational expectations

**DOI:** 10.3389/fpsyg.2026.1761912

**Published:** 2026-07-06

**Authors:** Huanran Liu, Huijuan Tian, Ding Wang, Anni Hu

**Affiliations:** 1 Education College, Hubei University, Wuhan, China; 2 Chibi No.1 Middle School, Xianning, China

**Keywords:** family background, junior high school students, learning engagement, parental educational expectations, self-educational expectations

## Abstract

**Introduction:**

Family background correlates with academic performance and attainment of junior high school students, with learning engagement acting as a mediator. Shifting focus from final academic outcomes to learning processes helps explore the internal mechanism linking family background and academic competence.

**Methods:**

This study established a parallel mediation model based on samples from two junior high schools in Central China. Family background was the independent variable, parental educational expectations and students’self-educational expectations served as parallel mediators, and learning engagement was the dependent variable.

**Results:**

After controlling for gender, grade and school type, family background showed a significant positive correlation with students’learning engagement. Students’self-educational expectations functioned as a mediator between family background and learning engagement, whereas the indirect association of parental educational expectations was not statistically significant.

**Discussion:**

The findings clarify the mechanism behind the association between family background and learning engagement among Chinese junior high school students, and provide practical references for peers in similar situations. Recommended practices include encouraging parental involvement, creating a harmonious family atmosphere, setting reasonable educational expectations, and regulating internal and external motivation to cultivate students’learning engagement.

## 1 Introduction

Some studies have shown that since China’s reform and opening-up, inequality in the distribution of educational opportunities in China has gradually intensified, and family background has become more strongly correlated with educational access (Li and Qiu, 2018). In particular, the educational level and professional status of parents play an important role (Mahadevan and Fan, 2021). With the development of society and the expansion of education scales, people are increasingly inclined to pursue higher-quality education. There is a correlation between family background and school quality (Li and Qiu, 2018), and students from advantaged family backgrounds are more likely to enter key schools (Tompsett and Knoester, 2023). Family background is generally measured as family capital and further concretized into family economic, cultural, and social capital. The direct association of Chinese family capital on children’s education has demonstrated an obvious upward trend (Ling and Zheng, 2018), and the advantages of family economic, cultural, and social capital contribute to people’s selection of superior educational resources, thus relating to their academic achievements (Zhao et al., 2023).

However, prior explorations of the relationship between educational attainment and family background have identified only superficial correlations. Students’ ultimate access to educational resources directly depends on their sustained learning engagement and family background. Their daily behaviors and family background are closely associated with the academic performance of junior high school students. Students’ participation in learning reflects the quality of the educational process, which constitutes the core of educational quality (Kuh, 2009). Therefore, investigating the relationship between family background and students’ learning engagement can deepen the understanding of the intrinsic connections between family background and educational quality, as well as the inherent mechanisms closely related to academic performance and educational attainment.

Across different educational stages, the learning engagement and academic performance of junior high school students strongly predict individuals’ future educational attainment and career success. Researchers have noted that educational performance at various stages ultimately accumulates to predict higher education admission opportunities, with primary and junior high schools making the most significant contributions to this cumulative process (Michaelowa, 2007). Lyu et al. (2019) found that family background strongly correlates with junior high school graduates’ access to key high schools. Taking China as a case study, this study investigates the relationship between family background and students’ learning engagement. The findings contribute to understanding the underlying mechanism through which family background is positively associated with learning engagement among some Chinese junior high school students. They also provide important implications for the learning outcome and academic achievement of junior high school students in similar situations.

## 2 Literature review and research hypotheses

### 2.1 Relationship between family background and junior high school students’ learning engagement

Student engagement in learning, also known as “student engagement” or “student involvement,” refers to students’ active participation in various educational activities in schools. Initially, it mainly referred to behavioral participation; later, psychological participation was considered, reflecting the variables in which students invest physical and mental energy in activities related to their studies (Astin, 1984). Student participation is categorized as behavioral, cognitive, and emotional (Pietarinen et al., 2014). Researchers have increasingly focused on junior high school students’ learning engagement, examining their participation status and the resources and conditions provided by universities (Zheng et al., 2024). Scholars in China have developed the Chinese College Student Engagement Scale to measure the quality of higher education in China or evaluate the quality of undergraduate teaching, drawing on relevant foreign research on junior high school students’ engagement in learning (Xu et al., 2023).

Factors predicting junior high school students’ learning engagement include their family background, interactions with faculty, and college conditions. Among these, family background is a crucial factor (Nguyen, 2019). The characteristics and environments of colleges and universities are crucial factors in student engagement. However, after controlling for school characteristics and demographic variables, subjective family background is more strongly associated with the individual process of higher education (Adeyeye and Dasoo, 2024). From an adaptability perspective, some researchers have noted that students with rich family capital show greater academic adjustment, which partly reflects family cultural capital and the intergenerational transfer of social capital in academic adjustment (Jin et al., 2024a). Previous studies have shown that students from wealthier families are associated with a more favorable pre-university status and expectations, thereby facilitating a smoother path to academic success (Qiu and Ye, 2023).

Few studies have directly explored the association between family background and academic engagement among junior high school students, though this topic has been touched upon in research on correlates of student engagement. Existing evidence indicates that family socioeconomic status is significantly positively correlated with adolescents’ academic engagement, and perceived social support serves as a full mediator between the two variables (Song et al., 2025). Other studies indicate that educational values and parental educational input are associated with students’ learning engagement, and that higher parental educational input correlates with greater academic engagement of their children (Liu, 2024). For example, the Coleman Report found that a family’s socioeconomic status is the main factor accounting for differences in students’ academic achievement. The status acquisition model proposed by Blau and Duncan also indicates a correlation between a family’s economic capital and children’s educational achievement (Dufur et al., 2013). Family socioeconomic status is a key variable in students’ educational achievement (Dufur et al., 2013). Therefore, children from higher socioeconomic status families are likely to achieve higher academic outcomes because they can purchase high-quality educational resources, attend high-performing schools, and access market-based educational services (Zhao et al., 2023).

Familial cultural capital also correlates closely with children’s academic achievement. Bourdieu’s cultural capital theory highlights its decisive role in academic achievement and personal development. By investigating the family situations and test scores of junior high school students, some researchers have found that cultural capital appears to be a mechanism for ensuring educational advantages for children from socially advantaged families (Potter and Roksa, 2013). Family cultural capital is an important factor in children’s schooling, as parents pass on language or cognitive skills to their children (Graaf et al., 2000). Most researchers agree that there is a strong link between educational opportunities and cultural capital (Jin et al., 2022), while others argue that family cultural capital is the most prominent factor correlating closely with children’s access to education, and that its correlation is stronger than that of family economic capital (Reay, 2004). Family cultural capital is divided among parents and children, and children can internalize their parents’ cultural capital, gaining advantages in their studies (Jin et al., 2024b).

Numerous studies have revealed a correlation between family social capital and children’s academic achievement. Different researchers exhibit distinct disparities in their understanding and operationalization of social capital. It is mainly measured from perspectives such as parents’ interpersonal networks, political affiliations (whether they are party members), occupations (whether they are employed in party and government institutions), parenting behaviors, educational expectations, and educational participation (Tan and Fang, 2023). However, this study examined social capital in the family context, which primarily refers to relationships within the family. The better the relationship between parents and children, the more stable the mirroring system between them becomes, enabling them to understand each other’s actions and intentions and to share emotions and feelings through “empathy.” This allows junior high school students to fully comprehend their parents’ intentions and emotions in social settings, thereby making successful decisions (Frith and Singer, 2008). Therefore, when a family possesses greater social capital, junior high school students are more likely to align with their parents’ intentions and devote more effort to their academic studies.

Overall, the academic performance of junior high school students is directly associated with their family background. Given that academic performance is closely linked to learning engagement, it serves as a crucial intermediary between family background and academic achievement. Consequently, by integrating studies on the relationship between family background and junior high school students’ learning engagement, as well as junior high school students’ academic performance, this paper proposes Hypothesis 1: Family background correlates closely with junior high school students’ learning engagement.

### 2.2 Indirect role of self-educational expectations

Intermediate processes between family background and learning engagement have not been fully explored; therefore, this study aims to examine these self-educational expectations.

As early as the introduction of the status acquisition model, some researchers expressed skepticism and proposed incorporating a socio-psychological variable, namely educational expectations. They noted that educational expectations serve as an intermediary variable linking family background, educational attainment, and career achievement. Furthermore, educational expectations are associated with family background and are simultaneously most strongly associated with educational attainment (Fuqin and Yiwen, 2014).

Whether a person chooses to commit to a particular task depends on their belief in their ability to accomplish it and the value they perceive in doing so. When an individual has high expectations for the task and believes they can accomplish it, they are more likely to commit to it (Eccles and Wigfield, 2024).

In addition, Academic Self-Concept Theory suggests that individuals’ perceptions of their academic competence correlate with their level of academic engagement. When students form relatively positive perceptions and evaluations of their academic abilities within academic contexts, they tend to develop higher self-educational expectations, which in turn are associated with students’ positive learning engagement behaviors (Song and Wang, 2026). Researchers have conducted a comprehensive study of the relationship between academic self-concept and academic achievement from a developmental perspective, noting that academic achievement is a significant predictor of academic self-concept (Wu et al., 2021). Academic self-concept, in turn, translates into self-educational expectations, and improvements in academic achievement are directly linked to the level of academic engagement.

Therefore, educational expectations are a powerful psychological motivator for adolescents to actively pursue academic success (Johnston et al., 2021). Some researchers have argued that students’ self-educational expectations are key to realizing “hope for the future,” and that they mediate the relationship between a family’s background and their children’s academic performance (Khattab, 2015). Under normal circumstances, parents’ educational levels and family income predict adolescents’ educational expectations (Pinquart and Ebeling, 2020). Children’s educational ambition is higher when their parents have more assets, and educational ambition mediates the relationship between parental assets and children’s academic achievement (Zhan, 2006). Researchers have demonstrated that individual educational expectations are important intermediary mechanisms. The higher the socioeconomic status of the students’ parents, the higher their expectations to attend college. Moreover, compared with children from households with lower college attendance expectations, those from households with higher socioeconomic status are far more likely to pursue higher education (Tompsett and Knoester, 2023).

High educational expectations among junior high school students increase their desire for education. An empirical analysis of the graduation of junior high school students in a county in Gansu Province, China, reveals that family economic capital is indirectly associated with academic achievement (Liu et al., 2020). Based on data from the China Education Tracking Survey, another empirical study on the association between self-educational expectations and the development of students demonstrates that, as a socio-psychological factor, junior middle school students’ self-educational expectations relate to educational outcomes and serve as an intermediary factor linking family background to students’ educational and career achievement (Garg et al., 2002). Generally, family background is closely related to a student’s personal development, and their educational expectations play an important indirect role in this association (Garg et al., 2002).

As is evident in the extant research, students’ educational expectations serve as a indirect variable in their professional and personal development. However, before students achieve good educational outcomes, their academic engagement must improve. Students’ academic engagement serves as a mediator between their educational expectations and academic achievement. The higher their educational expectations, the more likely they are to be fully engaged in the learning process, devote more effort, and suppress negative behaviors (Wigfield and Eccles, 2000). Therefore, in conjunction with the above-mentioned related studies, this study proposes Hypothesis 2: The educational expectations of junior high school students mediate the relationship between family background and academic engagement.

### 2.3 Indirect role of parental educational expectations

The above discussion indicates that an individual’s subjective expectations, aspirations, and corresponding behaviors closely relate to their academic performance. However, within a family, parents’ expectations can also be associated with their children’s academic performance (Seginer, 1983). In general, parents with higher expectations for their children are more likely to set higher standards for their children’s schooling and social interactions (Eccles and Harold, 2013). For example, Smith et al. found that parents’ expectations regarding their children’s performance can predict the location and level of their children’s later college attendance (Smith et al., 1995). Chinese researchers have also observed that parental educational expectations are important predictors of children’s educational attainment (Yamamoto and Holloway, 2010).

Parents’ educational expectations sometimes manifest as parenting styles. The higher their educational expectations, the more engaged they are in their children’s studies (Hoover-Dempsey and Sandler, 1995). Lareau and Weininger (2003) call this process “collaborative cultivation,” wherein parents pass on to their children “academic skills” that are critical to academic success (Lareau and Weininger, 2003). According to Lareau (2002), parents from socially advantaged backgrounds have higher psychological expectations and adopt a “collaborative cultivation” parenting style. Disadvantaged parents tend to have misjudgments and underestimate their children’s academic success (Yan and Gai, 2022). Bourdieu (2020) called this “habitus,” a structure of the mind that simultaneously reflects a certain social structure. Parents’ educational expectations correlate closely with their family background. The higher the parents’ socioeconomic status, the higher their educational expectations for their children (Davis-Kean, 2005). The higher the occupational status and educational level of the parents, the higher their educational expectations for their children (Tompsett and Knoester, 2023). The lower a family’s socioeconomic status, the lower the parents’ psychological expectations and confidence in their children’s education (Keijer, 2021). For example, many rural parents, based on their judgment of objective facts, believe that their children have no prospects for academic success (Cutler White and Chapman, 2024).

There is also a strong link between parental educational expectations and children’s expectations. The higher the educational expectations of parents, the more they help their children uphold and fulfill their own educational expectations (Yin et al., 2025), and show a significant positive correlation with their learning engagement and academic achievement (Pinquart and Ebeling, 2020). However, due to cultural values and significant others, parental and adolescent educational expectations are not always consistent. The two constructs show no direct linkage, and their correlations with academic performance also differ.

For instance, some scholars note that parental and adolescent educational expectations may converge or diverge. Adolescents’ self-expectations stand as a key factor for academic success and act as a mediator between family background and academic performance. Parental educational expectations interact with adolescents’ self-expectations during this process, forming an interactive relationship (Yin et al., 2025).

Combined with prior studies, parental and adolescents’ educational expectations are closely connected yet relatively independent, and both are strongly associated with family background. As a psychological state, adolescents’ self-expectations relate directly to positive learning engagement. Parental educational expectations correspond to parental educational involvement, which also shows positive correlation with adolescents’ learning engagement. Accordingly, Hypothesis 3 is proposed: Parental educational expectations serve as a mediator between family background and junior high school students’ learning engagement.

**Figure 1:**
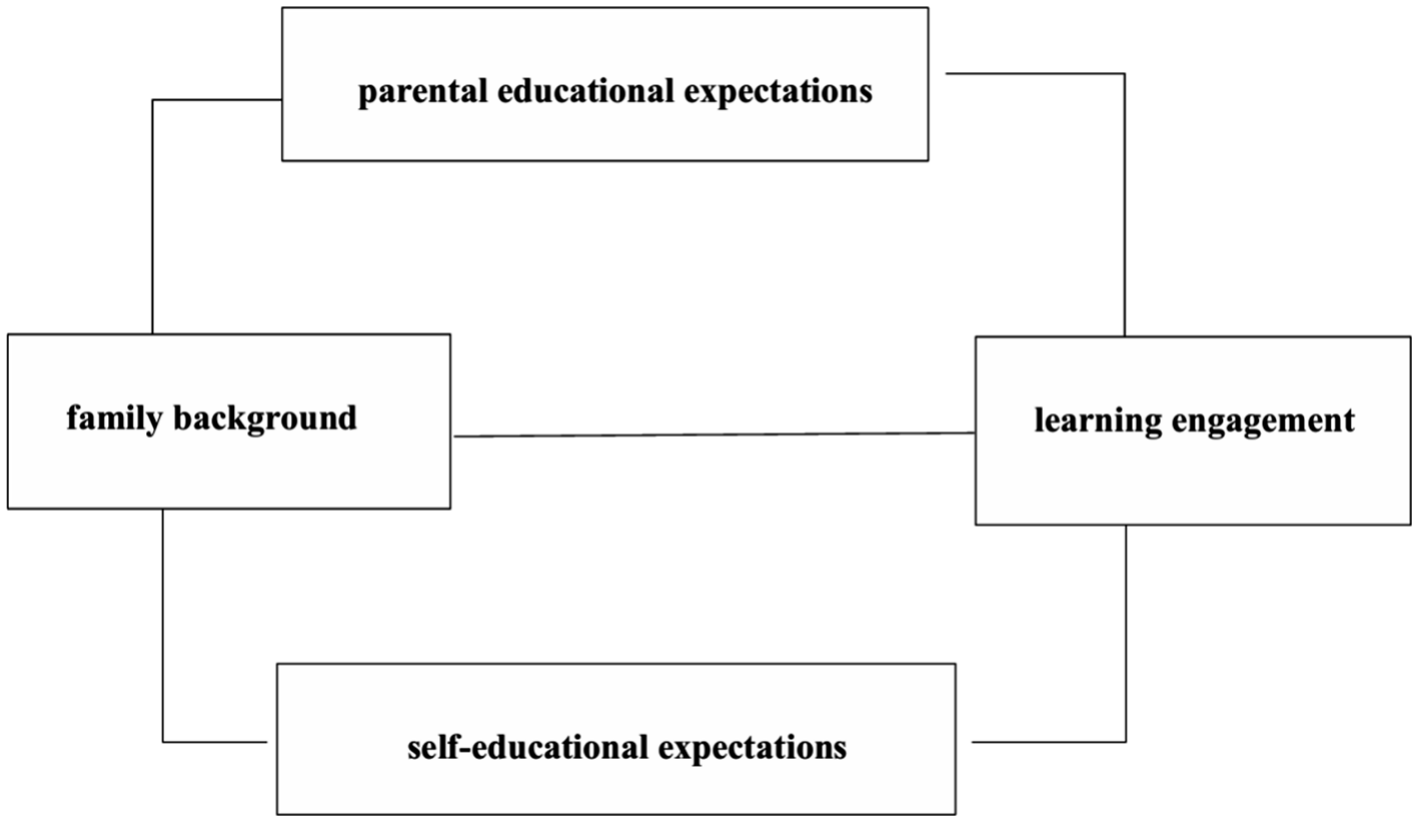
Analytical model for this study.

To sum up, this study established a dual mediation model to examine associations between family background and junior high school students’ learning engagement, and tested the indirect roles of self-educational expectations and parental educational expectations (Figure 1).

## 3 Materials and methods

### 3.1 Participants

This study was conducted in two junior high schools in central China: one private and the other public. The two schools are in the same district and city. The researchers used systematic sampling to select 845 students for participation in the study.

Online and offline questionnaires were distributed to the selected participants. All 845 questionnaires were recovered, of which 783 (92.66%) were valid. 459 (58.62%) participants were male students, and 324 (41.38%) were female students. Regarding grade distribution, there were 482 first-year students (61.56%), 208 second-year students (26.56%), and 93 third-year students (11.88%). Verbal informed consent was obtained from all participants. As per the “Measures for the Ethical Review of Biomedical Research Involving Human Beings” and relevant academic ethics norms, ethical review and approval were not required for this cross-sectional questionnaire survey, as no operations were undertaken that might harm the rights, physical and mental health, or privacy of the research participants.

### 3.2 Research methods and tools

In this study, the researchers used a questionnaire to survey students through a combination of online and offline methods. The questionnaires used a 5-point Likert scale, from 1 (strongly disagree) to 5 (strongly agree), with respondents rating their actual situations. Numerous theoretical studies have examined the relationship between family background and junior high school students’ learning engagement. Each variable is measured on a three-point scale: Family Background Scale, the Educational Expectation Scale, and the Learning Engagement Scale, respectively. These scales were designed by referring to mature scales and modifying them according to research needs.

#### 3.2.1 Family background scale

Family background can be classified into subjective and objective dimensions. In this study, family background was divided into three dimensions: economic, educational, and social.

To quantify the family’s economic background, this study used the parents’ monthly income. For the questionnaire item measuring parents’ monthly household income, we defined this variable as the sum of the father’s and mother’s monthly incomes, and established five categorical levels as follows: below 8,000 yuan, 8,000–16,000 yuan, 16,000–24,000 yuan, 24,000–32,000 yuan, and above 32,000 yuan. We analyzed the questionnaire data and found that the primary parents’ monthly household income bracket for public school students was 8,000–16,000 yuan, accounting for 43% of the public school sample; by contrast, we identified the dominant bracket for private school students as 16,000–24,000 yuan, which represents 24% of the private school sample.

Family educational background was assessed based on the parents’ educational levels and their involvement in their students’ education. For the family educational background dimension, we designed three questionnaire items: father’s educational attainment, mother’s educational attainment, and parental educational involvement. We established five categorical levels of parents’ educational attainment, including primary school, junior high school, senior high school, and others. Correspondingly, we set five hierarchical levels for parental educational involvement, with representative statements such as “Your parents pay close attention to your academic performance.” The Cronbach’s *α* reliability coefficient for the family educational background construct was 0.755.

Family social background was assessed based on the social relationship between the parents and students. For the family social background dimension in the questionnaire, we designed four items, each with five-point Likert scale response options. A representative example is the item: “Your parents allow you to hold opinions different from theirs,” with response options including “Agree,” “Somewhat agree,” “Strongly agree,” “Somewhat disagree,” and “Strongly disagree.” Through questionnaire data analysis, we found that the Cronbach’s α reliability coefficient for the family social background construct was 0.855.

#### 3.2.2 Educational expectations scale

The Parental Educational Expectations Scale consists of a single item: “What is the highest educational level your parents expect you to attain?” with five response options: junior high school, senior high school, bachelor’s degree, master’s degree and doctoral degree. The Self-Educational Expectations Scale also includes one item: “What is the highest educational level you expect to attain?,” with identical five options.

Educational expectations here are gauged by educational attainment via single-item measures. Though such measures differ from multi-item scales in accuracy, they feature simplicity, low respondent burden and high applicability for time- and resource-limited research, and can be comparable to multi-item measurements (Pandit et al., 2026). The construct of educational expectations has a clear and focused connotation, referring to individuals’ or others’ quantitative judgment on future educational attainment. Single-item measurement is appropriate for concrete, unidimensional constructs with unambiguous meaning for respondents (Pinquart and Ebeling, 2020). The Cronbach’s alpha coefficient for the educational expectations construct is 0.642.

#### 3.2.3 Learning engagement scale

The degree of learning participation is defined as an evaluation that considers students as the object, with students’ cognition, behavior, and emotions serving as the evaluation content, and students’ self-evaluation as the main component. This represents a shift from performance-oriented teaching to focusing on learning processes. In this study, a learning engagement scale was developed based on existing scales. The evaluation was conducted based on three aspects: behavioral, cognitive, and emotional participation. Among all questionnaire items, we included three items for the Learning Engagement Scale, corresponding to three dimensions: Behavioral Engagement, Cognitive Engagement, and Emotional Engagement. For the Behavioral Engagement item, we designed four response options, such as “I carefully preview courses before class.” For the Cognitive Engagement item, we also set four response options, including “I prefer to explore answers independently rather than receive direct solutions.” For the Emotional Engagement item, we developed seven response options, exemplified by “I actively participate in school activities because I am interested in them.” The Cronbach’s *α* reliability coefficient for the learning engagement construct was 0.904.

### 3.3 Scale reliability test

The Cronbach’s α coefficient was calculated using the reliability analysis function in SPSS based on the 783 valid questionnaires recovered. For further verification, Cronbach’s alpha coefficient of each scale was calculated separately. The reliability coefficients were as follows: family background scale, 0.826; family educational background scale, 0.755; family social background scale, 0.855. Both the overall reliability of the questionnaire and that of each dimension were satisfactory, indicating that the survey results are reliable.

Confirmatory factor analysis was implemented for the measurement model of family background to examine overall model fit. Fit indices were reported as CFI = 0.931, TLI = 0.903, RMSEA = 0.086 (90%CI: 0.078–0.095), and SRMR = 0.090. CFI and TLI exceeded the conventional cutoff of 0.90, while RMSEA and SRMR fell within acceptable ranges. The well-fitted measurement model supports subsequent structural path evaluation.

Meanwhile, this study also conducted confirmatory factor analysis on the measurement model of the learning engagement dimension to verify the overall fit of the model. The results showed that the GFI was 0.927, the TLI was 0.910, the SRMR was 0.052, and the RMSEA was 0.078 (90%CI: 0.071 ~ 0.085). All the indicators met the standards, and the reliability was acceptable.

To examine whether common method bias was present in this study, we analyzed the data using Harman’s one-factor test. The results showed that the first common factor explained 27.50% of the variance (less than 40%), indicating that the scales used in this study did not exhibit serious common method bias.

To validate the questionnaire’s structural validity, exploratory factor analysis was conducted. The statistical results indicated that the Kaiser–Meyer–Olkin (KMO) sampling adequacy test yielded a value of 0.901, exceeding 0.9, suggesting that the data were highly suitable for factor analysis. Bartlett’s test statistic was 10586.150, and *p* < 0.001. Moreover, the communalities of all the research items were higher than 0.4. Overall, the questionnaire’s validity was satisfactory.

## 4 Research results

### 4.1 Correlation analysis of self-education expectations, parental education expectations, family background, and learning engagement

Table 1 presents the Pearson correlation results of four variables: self-educational expectations, parental educational expectations, family background and learning engagement. All variables yield significant positive correlations (*p* < 0.01). The correlation coefficient between learning engagement and family background ranks the highest (*r* = 0.583, *p* < 0.01), reflecting a strong positive association. The pair of self-educational expectations and parental educational expectations shows the second-highest coefficient (*r* = 0.473, *p* < 0.01), with a moderate positive association. Significant weak-to-moderate positive associations are also found between self-educational expectations and learning engagement (*r* = 0.246, *p* < 0.01$), parental educational expectations and learning engagement (*r* = 0.239, *p* < 0.01), parental educational expectations and family background (*r* = 0.215, *p* < 0.01), and self-educational expectations and family background (*r* = 0.173, *p* < 0.01).

**Table 1:** Correlation analysis of self-education expectations, parental education expectations, family background, and learning engagement.

	Self-education expectations	Parental education expectations	Learning engagement	Family background
Self-education expectations	1	0.473**	0.246**	0.173**
Parental education expectations,	0.473**	1	0.239**	0.215**
Learning engagement	0.246**	0.239**	1	0.583**
Family background	0.173**	0.215**	0.583**	1

*p* < 0.01 indicates that the correlation is significant at the 0.01 level (two-tailed).

### 4.2 Scatter plot analysis of family background and learning engagement

**Figure 2:**
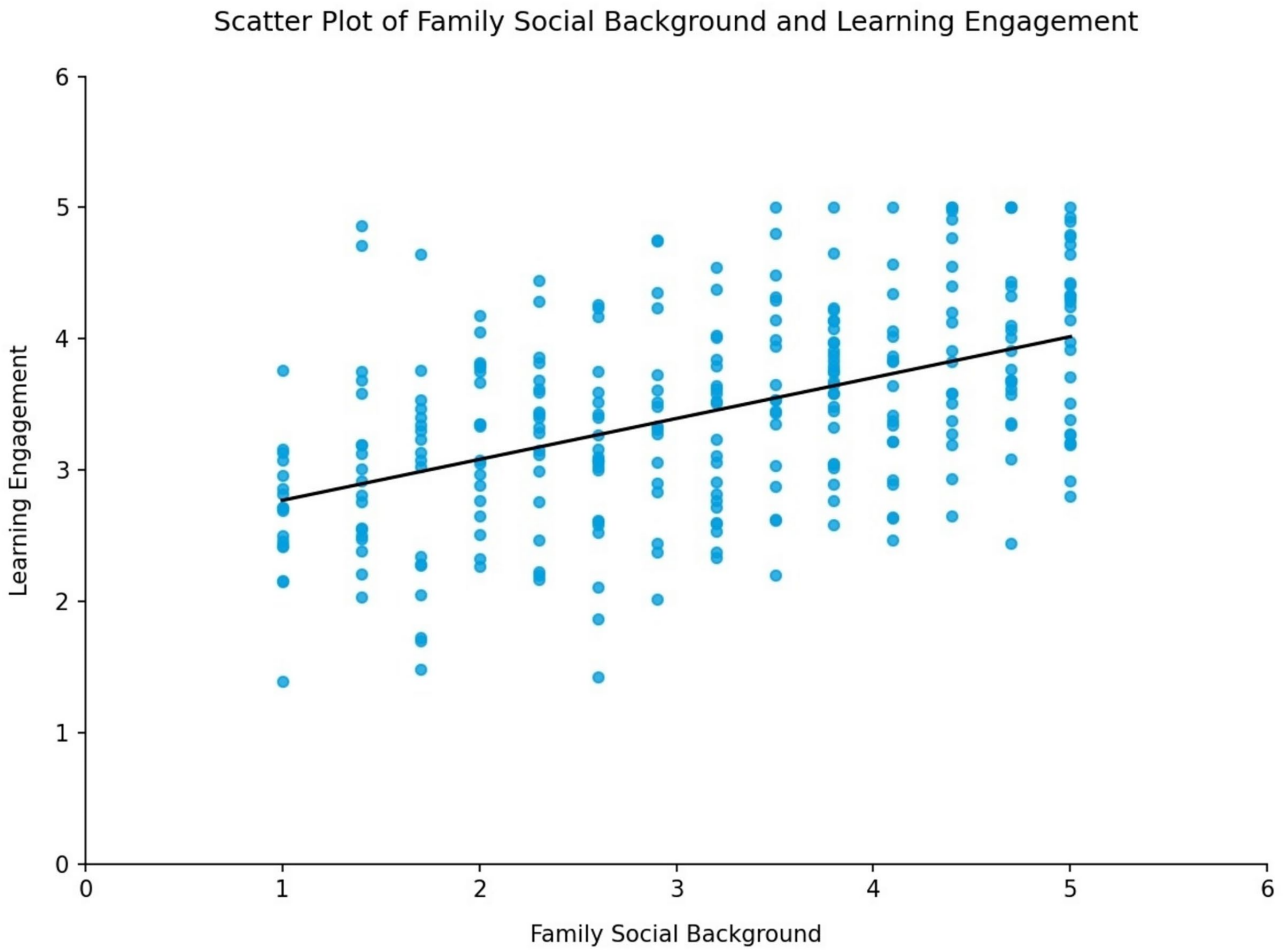
Scatter plot of family social background and learning engagement.

This study plotted a scatter graph and a fitted trend line of the family social background and the learning participation of junior high school students, to visually present the characteristics of their relationship. From the graph, as the level of family social background increases, the overall learning participation of junior high school students shows a significant upward trend. The slope of the fitted regression line is positive, indicating a significant positive correlation between the two, that is, the better the family social background, the higher the level of learning participation of junior high school students. Meanwhile, the distribution of the scattered points is relatively uniform, and no obvious outliers or extreme deviations are present, indicating that the data distribution is relatively stable and there is no serious heteroscedasticity. This further verifies the reliability of the relationship and corroborates the results of the previous regression analysis (Figure 2).

### 4.3 Analysis of indirect association

This study adopted Hayes’ (2022) SPSS PROCESS macro (Model 4) to examine the dual mediation of self-expectations and parental educational expectations between family background and learning engagement among junior high school students. Grade, gender and school type were included as covariates. The Bootstrap method with 5,000 resamples was used to compute 95% confidence intervals; an interval excluding zero indicates statistical significance. Descriptive statistics and correlation matrix of all variables are presented in Table 2, regression results in Table 3, and mediation test results in Table 4.

**Table 2:** Descriptive statistics and correlation coefficient matrix of each variable (*N* = 783).

Variable	*M*	SD	1	2	3	4	5	6
1. Family background	42.53	8.22	–					
2. Self-education expectations	3.97	1.03	0.1733**	–				
3. Parental education expectations	4.08	0.99	0.2151**	0.4732**	–			
4. Learning engagement	53.93	11.39	0.5829**	0.2458**	0.2386**	–		
5. Grade	1.50	0.70	−0.0578	−0.0771*	−0.1105**	−0.1672**	–	
6. Gender	1.41	0.49	−0.0316	0.0336	0.0729*	−0.0852*	0.0147	–
7. School type	1.69	0.46	0.1437**	0.0926**	0.1056**	0.0460	0.0964**	−0.0625

M, mean; SD, standard deviation; Gender is coded as 1 for male and 2 for female; School type is coded as 1 for urban and 2 for rural. *p < 0.05. **p < 0.01.

**Table 3:** Regression coefficients of each path in the mediating model.

Dependent variable	Independent variable	*β*	SE	*t*	*p*	95% CI
Self-education expectations						
	Constant	2.8554	0.2589	11.0310	<0.001	[2.3473, 3.3635]
	Family background	0.0199	0.0045	4.4661	<0.001	[0.0112, 0.0287]
	Grade	−0.1125	0.0521	−2.1589	0.0312	[−0.2149, −0.0102]
	Gender	0.0935	0.0735	1.2721	0.2037	[−0.0508, 0.2379]
	School type	0.1787	0.0799	2.2368	0.0256	[0.0219, 0.3354]
Parental education expectations						
	Constant	2.7129	0.2446	11.0896	<0.001	[0.0096, 0.0424]
	Family background	0.0238	0.0042	5.6537	<0.001	[0.0156, 0.0321]
	Grade	−0.1547	0.0493	−3.1397	0.0018	[−0.2514, −0.0580]
	Gender	0.1736	0.0695	2.4982	0.0127	[0.0372, 0.3100]
	School type	0.1997	0.0755	2.6455	0.0083	[0.0515, 0.3479]
Learning engagement						
	Constant	20.7360	2.5312	8.1922	<0.001	[15.7672, 25.7048]
	Family background	0.7569	0.0404	18.7243	<0.001	[0.6776, 0.8363]
	Self-education expectations	1.3224	0.3546	3.7295	<0.001	[0.6263, 2.0184]
	Parental education expectations	0.7171	0.3752	1.9114	0.0563	[−0.0194, 1.4536]
	Grade	−1.8621	0.4651	−4.0039	<0.001	[−2.7750, −0.9491]
	Gender	−1.7922	0.6542	−2.7396	0.0063	[−3.0764, −0.5080]
	School type	−1.0863	0.7115	−1.5267	0.1272	[−2.4830, 0.3105]

R2 (self-education expectation) = 0.0423, *F*(4,778) = 8.5909, *p* < 0.001; R2 (parental education expectation) = 0.0708, *F*(4,778) = 14.8263, *p* < 0.001; R2 (learning engagement) = 0.3863, *F*(6,776) = 81.4026, *p < 0.001.

**Table 4:** Bootstrap indirect association test results.

Path	Indirect association	BootSE	BootLLCI	BootULCI	Standardized indirect association
Total indirect association	0.0434	0.0125	0.0212	0.0705	0.0313
Family background → Self-education expectations → Learning engagement	0.0263	0.0106	0.0092	0.0501	0.0190
Family background → Parents’ educational expectations → Learning engagement	0.0171	0.0107	−0.0031	0.0392	0.0123
Direct association	0.7569	0.0404	0.6776	0.8363	0.5461

The number of Bootstrap sampling iterations = 5,000; If the 95% confidence interval (BootLLCI, BootULCI) does not include 0, it indicates a significant association.

#### 4.3.1 Descriptive statistics and correlation analysis

Table 2 presents the means, standard deviations and Pearson correlation coefficients of all variables. Family background is positively correlated with learning engagement (*r* = 0.5829), (*p* < 0.01), self-educational expectations (*r* = 0.1733), (*p* < 0.01) and parental educational expectations (*r* = 0.2151, *p* < 0.01). Positive correlations are also observed between self-educational expectations, parental educational expectations and learning engagement (*r* = 0.2458, 0.2386, *p* < 0.01). A moderate positive correlation exists between self-educational expectations and parental educational expectations (*r* = 0.4732, *p* < 0.01).

#### 4.3.2 Regression path analysis

To examine the relationships along each path, three regression equations were constructed, with results summarized in Table 3.

Regression with self-educational expectation as dependent variable: With family background, grade, gender, and school type as predictors, the overall model was significant [*F*(4, 778) = 8.5909, *p* < 0.001], adjusted *R*^2^ = 0.0423. Family background showed a significant positive association with self-educational expectations (*β* = 0.0199, SE = 0.0045, *t* = 4.4661, *p* < 0.001, 95% CI [0.0112, 0.0287]). Among control variables, grade was significantly negatively associated (*β* = −0.1125, *p* = 0.0312), school type significantly positively associated (*β* = 0.1787, *p* = 0.0256), while gender was not significantly associated (*p* = 0.2037).

Regression with parental educational expectation as dependent variable: The overall model was significant [*F*(4, 778) = 14.8263, *p* < 0.001], adjusted *R*^2^ = 0.0708. Family background showed a significant positive association with parental educational expectations (*β* = 0.0238, SE = 0.0042, *t* = 5.6571, *p* < 0.001, 95% CI [0.0156, 0.0321]). Among controls, grade was significantly negatively associated (*β* = −0.1547, *p* = 0.0018), gender significantly positively associated (*β* = 0.1736, *p* = 0.0127), and school type significantly positively associated (*β* = 0.1997, *p* = 0.0083).

Regression with learning engagement as dependent variable: Family background, self-educational expectations, parental educational expectations, and the three control variables were entered simultaneously. The overall model was significant [*F*(6, 776) = 81.4026, *p* < 0.001], adjusted *R*^2^ = 0.3863. Family background showed a significant direct association with learning engagement (*β* = 0.7569, SE = 0.0404, *t* = 18.7243, *p* < 0.001, 95% CI [0.6776, 0.8363]). Self-educational expectations were significantly positively associated with learning engagement (*β* = 1.3224, SE = 0.3546, *t* = 3.7295, *p* = 0.0002, 95% CI [0.6263, 2.0184]). However, the association between parental educational expectations and learning engagement did not reach statistical significance (*β* = 0.7171, SE = 0.3752, *t* = 1.9114, *p* = 0.0563, 95% CI [−0.0194, 1.4536]). Among control variables, grade (*β* = −1.8621, *p* = 0.0001) and gender (*β* = −1.7922, *p* = 0.0063) were significantly negatively associated with learning engagement, whereas school type was not significantly associated (*p* = 0.1272).

#### 4.3.3 Testing of indirect association

The significance of indirect association was examined using the bootstrap method, with results presented in Table 4. The total indirect association between family background and learning engagement was significant (indirect association = 0.0434, BootSE = 0.0125, 95% CI [0.0212, 0.0705]). Specifically, the indirect pathway via self-educational expectations was significant (indirect association = 0.0263, BootSE = 0.0106, 95% CI [0.0092, 0.0501]), with a confidence interval that did not include zero. In contrast, the indirect pathway via parental educational expectations was not significant (indirect association = 0.0171, BootSE = 0.0107, 95% CI [−0.0031, 0.0392]), as its confidence interval contained zero.

#### 4.3.4 Summary

Synthesizing the above results, self-educational expectations served as a significant partial mediator between family background and junior high school students’ learning engagement, whereas the mediating role of parental educational expectations did not reach statistical significance. These findings indicate that family background is associated with learning engagement both directly and indirectly through higher levels of self-educational expectations. The total association between family background and learning engagement (direct + indirect) was 0.8003, with the direct component accounting for approximately 94.6% and the indirect component for approximately 5.4%.

## 5 Discussion

### 5.1 Family background is directly associated with junior high school students’ learning engagement

After controlling for variables such as school type and individual heterogeneous traits, this study tests the hypothesis 1 regarding the association between family background and junior high school students’ learning engagement. The associations of a family’s economic, educational, and social backgrounds differed. Family cultural and social capital are associated with students’ learning engagement, whereas family economic capital shows no association.

Cultural capital theory emphasizes the close correlation between cultural capital, school education, and students’ personal achievements. Families differ in the type and amount of cultural capital, and these differences constitute a key factor driving disparities in children’s academic performance (Jin et al., 2024b). In families with high cultural capital, parents leverage their advanced educational attainment to be associated with higher levels of their children’s learning and cognitive abilities, enabling the latter to better cope with school coursework. Furthermore, within a cultural environment correlated with cultural capital, parents engage in their children’s learning through practices such as “prioritizing academic performance,” “active collaboration,” “participating in school activities,” and “patient tutoring.” These practices equip children with superior learning experiences and stronger educational beliefs.

Family social capital is also closely associated with junior high school students’ learning engagement (Ye and Arifani, 2024). Social capital can be classified as internal or external (Hashavia-Tzuri et al., 2024). In this study, internal social capital was defined as intrafamilial social capital, consistent with previous studies that found intrafamilial social capital predicts students’ academic performance (Hashavia-Tzuri et al., 2024). This study further revealed that intrafamilial social capital predicts students’ learning engagement, characterized by emotionally warm interactions marked by understanding, encouragement, and respect (Tan and Fang, 2023). Warm interactions within the family enables children to experience positive emotions, leading to higher levels of learning engagement.

By contrast, this study found that family economic capital had no association on junior high school students’ academic engagement. In our main model, family background is correlated with learning engagement among junior high school students, whereas family economic capital as a component of family background does not predict their learning engagement. Previous studies exploring the relationship between economic capital, educational opportunities, and academic achievement have mostly found a correlation, indicating that the more parents can invest in their children’s education, the better the quality of educational resources that their children receive (Hango, 2007). People can utilize socioeconomic resources to place their children in key schools within the system and access better educational services for them (Zhao et al., 2023). In recent years, shadow education has become an important factor in enabling children from families with more financial resources to win educational competitions (Lu et al., 2023). Nevertheless, educational opportunities and access to educational resources in this context are all outcome variables, which can even be equivalently exchanged for economic capital. By contrast, learning engagement is a process variable that reflects educational status, and family economic capital exerted no significant association on it.

### 5.2 Self-educational expectations and learning engagement in junior high school students: the role of mediation

This study documents significant positive correlations between family background and students’ self-educational expectations (*β* = 0.0199, *p* < 0.001), and between such expectations and learning engagement (*β* = 1.3224, *p* < 0.001). Family background holds a direct positive linkage with junior high students’ learning engagement alongside an indirect association through self-educational expectations, with the indirect association markedly weaker than the direct linkage, supporting partial indirect association for self-educational expectations between the two variables.

Families endowed with superior cultural and social capital regularly supply academic guidance, sustain frequent parent–child communication, and form strong direct linkages with children’s learning engagement. This pattern renders the indirect association via self-educational expectations relatively marginal and places the direct linkage in a dominant position.

In fact, family background links to students’ self-educational expectations as well as their educational perceptions and academic choices. Pathways connecting family background and learning engagement may rely on holistic family educational strategies instead of self-educational expectations, resulting in a modest indirect association of such expectations. Even so, family background maintains correlations with self-educational expectations, which correspond to variations in junior high students’ learning engagement.

Households with abundant cultural and social capital feature frequent parental educational involvement and steady growth in children’s cognitive abilities and academic standing, contributing to elevated self-educational expectations. These expectations develop into internal psychological resources paired with active learning engagement. Early adolescence coincides with shifts from heteronomous to autonomous moral reasoning and advancing physical and mental development alongside rising self-awareness. Within supportive household surroundings, students set stricter personal standards, and inherent learning aspirations and educational expectations correspond to positive status in learning engagement.

### 5.3 Parental educational expectations and junior school students’ learning engagement: parallel mediation test

This study also identifies significant positive correlations between family background and junior high school students’ learning engagement (*β* = 0.7569, *p* < 0.001), as well as between family background and parental educational expectations (*β* = 0.0238, *p* < 0.001). Parental educational expectations show no statistical significance in the linkage with learning engagement (*β* = 0.7171, *p* = 0.0563), and the pathway via parental educational expectations is non-significant (indirect association = 0.0171, BootSE = 0.0107, 95% CI [−0.0031, 0.0392]).

Family background presents positive linkages with both parental educational expectations and students’ learning engagement. However, parental educational expectations are not linked to students’ learning engagement and show no indirect association between family background and learning engagement, failing to form a parallel pathway alongside students’ self-educational expectations.

A plausible explanation is that families with abundant capital resources engage directly in children’s education. Such direct behavioral support corresponds to higher learning engagement among students. Family background thus maintains a primary direct linkage with learning engagement, while parental educational expectations exhibit limited relevance in this framework.

Excessively high parental educational expectations may trigger stress and anxiety among adolescents, accompanied by negative behavioral patterns and reduced learning engagement. Excessively high expectations can also undermine students’ sense of competence, leading extrinsic motivation to override intrinsic motivation; motivation tends to fade once external pressure subsides.

As Ryan and Deci (2000), humans are innately inclined toward growth and self-actualization. Autonomy, competence and relatedness underpin well-being and proactive behavior. Social environments that fulfill these three basic psychological needs can sustain intrinsic motivation. Their work highlights the relevance of intrinsic motivation and competence to individual behaviors and long-term attainment, as well as the value of personal autonomy.

Conversely, overly low parental educational expectations are also unfavorable. They may induce negative self-fulfilling prophecies. Students may perceive a lack of parental confidence and develop learned helplessness, which correlates with passive learning engagement. External expectations shape individual behaviors implicitly. Students may internalize low parental expectations, alongside diminished self-perception and effort.

Hence, parental educational expectations fail to fully align with individual learning engagement. Despite the positive correlation between family background and parental educational expectations, no indirect association via parental educational expectations emerges between family background and learning engagement.

## 6 Conclusion

Unlike prior studies centering on the linkage between family background and school admission or academic attainment, this research targets in-school learning processes to examine factors and internal mechanisms tied to junior high students’ learning engagement.

Drawing on existing theories and empirical evidence, a parallel mediation model is constructed with family background as the independent variable, parental educational expectations and students’ self-educational expectations as parallel mediators, and learning engagement as the dependent variable. Analytic outcomes show a notable positive correlation between family background and students’ learning engagement. Self-educational expectations form an indirect association between family background and learning engagement, whereas such an indirect association is absent for parental educational expectations.

Derived from these findings, two sets of practical suggestions are formulated to support students’ learning engagement and subsequent academic and occupational attainment.

First, expand parental educational involvement and build harmonious family environments. Consistent with study results, family cultural and social backgrounds present notable correlations with junior high students’ learning engagement. Parents therefore prioritize children’s education and cultivate positive domestic cultural surroundings and harmonious interpersonal bonds within households.

Early adolescence marks a vulnerable and sensitive developmental period for youngsters. Parental respect, trust and encouragement deliver positive affective experiences and accumulate inner psychological resources. Equal and friendly parent–child interactions bring warmth and a sense of being valued, motivating adolescents to share academic matters and seek parental advice and backing. Such supportive domestic settings correspond to elevated self-educational expectations and consistent devotion to learning activities.

Second, establish appropriate parental educational expectations and adjust internal and external motivational drives concerning junior high students’ learning engagement. This study confirms an indirect association of students’ self-educational expectations linking family background and learning engagement, whereas no such indirect association emerges for parental educational expectations.

Higher self-educational expectations correspond to more favorable standing in students’ learning engagement, a trend unseen for parental educational expectations. Parents thereby set realistic expectations in line with children’s self-awareness and academic conditions to sustain elevated self-educational expectations. Despite non-significant linkage between parental educational expectations and learning engagement, appropriate parental educational expectations correlate with higher self-educational expectations. Elevated inner psychological resources derived from such expectations convert into concrete learning behaviors alongside greater learning engagement.

### 6.1 Limitations and future research directions

This study has several limitations that warrant future research, mainly in four aspects.

First, this study only explored the basic mechanism by which family background predicts junior high school students’ learning engagement, identifying the indirect role of self-educational expectations and the moderating role of parental educational expectations. However, other variables may also serve as mediators or moderators in this process.

Second, the sample selection neglected group differences among students. The study included only one public and one private school, leading to insufficient representativeness and limited exploration of group variations in learning engagement and its determinants.

Third, measurement items for parental and self-educational expectations were inadequate, failing to capture the full scope of the constructs and possibly distorting true relationships among variables, which may explain the weak indirect association of self-educational expectations. At the same time, the Cronbach’s *α* for the educational expectation scale (*α* = 0.642) falls below the conventional threshold of 0.70, which also represents a limitation of the present study.

Fourth, data for all variables were gathered via a single self-report questionnaire. This cross-sectional design presents limitations. Single-source data may entail common method bias. While this bias was verified to be mild, it could slightly alter the strength of correlations across variables. Social desirability bias and recall bias among respondents may also interfere with measurements of several variables.

Future studies should include more potential mediators and moderators, expand sample diversity, investigate group differences, and develop more rigorous and comprehensive measurement instruments. In addition, future research can further validate the stability of the conclusions of this study by collecting data from multiple sources (such as combining students’ self-assessments, teachers’ evaluations, and parents’ evaluations) or through longitudinal tracking designs.

## Data Availability

The original contributions presented in the study are included in the article/supplementary material, further inquiries can be directed to the corresponding author.
